# Quantification of 3,4-Dimethyl-1H-Pyrazole Using Ion-Pair LC–MS/MS on a Reversed-Phase Column

**DOI:** 10.1093/jaoacint/qsac126

**Published:** 2022-10-26

**Authors:** Gregory S Doran, Jason R Condon, Brooke F Kaveney

**Affiliations:** Gulbali Institute, Charles Sturt University, School of Agricultural, Environmental and Veterinary Sciences, Wagga Wagga, NSW 2678, Australia; Gulbali Institute, Charles Sturt University, School of Agricultural, Environmental and Veterinary Sciences, Wagga Wagga, NSW 2678, Australia; Gulbali Institute, Charles Sturt University, School of Agricultural, Environmental and Veterinary Sciences, Wagga Wagga, NSW 2678, Australia

## Abstract

**Background:**

Few methods exist for the analysis of the soil nitrification inhibitor 3,4-dimethyl-1H-pyrazole (3,4-DMP), which is a pesticide with the ability to reduce the production of nitrogenous greenhouse gases in soils as a result of fertilizer application. Due to its small size and polar nature, 3,4-DMP can be difficult to retain on an LC column, which makes diversion of a co-extracted soil matrix away from the MS/MS impossible.

**Objective:**

The current study aims to better control the retention time (RT) of 3,4-DMP. Additionally, 3,4-DMP-15N2 was synthesized and used as an internal standard for the soil extraction of 3,4-DMP.

**Methods:**

Perfluoroalkanoic acids were used as ion-pair reagents and were compared for their abilities to improve peak shape and RT, to better separate 3,4-DMP from the soil matrix without the need for cleanup during soil extraction.

**Results:**

RTs increased with both the carbon number and the concentration of the perfluoroalkanoic acid, and this improved peak shape and height. Perfluorooctanoic acid performed best, and improved peak height (PH) and shape were obtained by increasing the flow rate, resulting in a better S/N than from formic acid. The method provided a 10-fold improvement limit of quantitation on the most sensitive existing method and the use of 3,4-DMP-15N2 as an internal standard resulted in recoveries of 101–107%.

**Conclusion:**

Ion-pair reagents drastically increased the retention of 3,4-DMP and allowed the re-use of old LC columns that may otherwise be discarded. Improved separation of 3,4-DMP from the soil matrix allowed much of the matrix to be diverted from the MS/MS spray chamber.

**Highlights:**

Greater control of 3,4-DMP retention by the LC column resulting in the ability to separate 3,4-DMP from the soil matrix. The inclusion of ion-pair reagents only in the aqueous phase reduced ionization suppression of the analytes in the MS source.

Application of nitrogen fertilizers is essential for the broad scale farming required to meet the food needs of expanding global populations. Consequently, worldwide demand for nitrogen fertilizer increased from 120 million tonnes in 2018 (1) to a predicted 190 million tonnes in 2022 (2). Overuse of nitrogen fertilizers can be attributed to poor uptake efficiency of nitrate by plants coupled with the rapid biological conversion of ammonia to nitrate, leading to leaching from root zones. The environmental consequences include toxic algal blooms in surface water sources (3), as well as increased degradation of nitrates to nitrogen oxide gases, some of which are considered 300 times more potent as greenhouse gases than CO_2_ (4). Improved plant uptake efficiency of ammonia can result in lower application rates of nitrogen fertilizers, and is achievable by slowing ammonia conversion to readily leachable nitrates using nitrification inhibitors, such as 3,4-dimethyl-1H-pyrazole phosphate (3,4-DMPP). These pesticides can both block the microbial enzymes (5) responsible for the conversion, while also enhancing the ability of denitrifying bacteria to reduce N_2_O to the harmless N_2_ (6).

A sensitive analytical method is essential to detect trace pesticide residues applied to soil in order to ensure human safety, and to identify environmental accumulation of pesticide residues. To date, two methods have been published regarding the analysis of 3,4-dimethyl-1H-pyrazole (3,4-DMP), which is the active component of 3,4-DMPP. The first method required extensive sample cleanup and pre-concentration to adequately reduce optical interferences caused by soil organic matter (SOM; 7), while the second relied on limited cleanup for analysis by LC–MS/MS (8), and provided a LOQ than the former, while requiring 10 000 times less 3,4-DMP on-column. The method used 3,5-dimethyl pyrazole-^15^N_2_ (3,5-HDMP) as an internal standard, which performed consistently, but the 83–86% recovery indicated that it did not behave identically to the 3,4-DMP analyte. However, the analytical method failed to adequately retain 3,4-DMP on the LC column, resulting in its elution in a high mobile phase water concentration, while also failing to separate it from extracted SOM and limit SOM entry into the MS/MS spray chamber.

Ion pairing involves the inclusion to the LC mobile phase of a modifier that bears the opposite electrostatic charge as the analyte, and a non-polar tail region that allows increased interaction with the stationary phase to improve analyte retention and reduce band broadening that comes with the analysis of small, ionizable chemicals such as 3,4-DMP. The following work reports the use of ion-pair chromatography to better control the LC retention of 3,4-DMP, to improve peak shape and S/N, and includes the use of a ^15^N_2_ analogue of 3,4-DMP (3,4-HDMP) as an internal standard to attempt to find an internal standard that behaves more like 3,4-DMP.

## Experimental 

### Chemicals and Reagents

3,4-DMP (>98.0%), fluorinated alkanoic acids (C2–C8), 3-methyl-2-butanone, sulfuric acid, and sodium hydroxide were purchased from Sigma Aldrich (Sydney, Australia). Methanol and acetonitrile were supplied by Mallinckrodt (Sydney, Australia), and ^15^N_2_-hydrazine sulfate was manufactured by Toronto Research Chemicals, supplied by Sapphire Bioscience (Sydney, Australia).

### Ion-Pair Reagents

Perfluoroalkanoic acids (PFAAs) in the C2–C8 series were used as ion-pair reagents in the mobile phase at a concentration of 0.1% (w/v) in the water phase only, and compared to 0.1% formic acid which was used in the previous method (8). The PFAA concentration was reduced to 0.01% after the best-performing modifier was identified, and flow rate was varied to identify the best peak shape and retention time (RT) to allow separation of the 3,4-DMP from potential matrix interferences from soil extraction.

### Synthesis of 3,4-DMP-^15^N2 (3,4-HDMP)

Synthesis of 3,4-HDMP was based on an existing method (9) and scaled down more than 100-fold. ^15^N_2_-hydrazine sulfate (250 mg) was added to a round-bottom flask (15 mL) containing sulfuric acid (350 µL, 70%), sodium iodide (10 mg,) and a magnetic stirrer bar. 3-Methyl-2-butanone (150 µL) was added slowly and the mixture heated and stirred under side-arm reflux (125°C, 1 h). After cooling, sodium hydroxide (2.5 mL, 5 M) was added and the solution was extracted with toluene (3 × 2 mL). Toluene was evaporated to dryness under nitrogen gas (25°C) and the residue resuspended in acetonitrile (1 mL) for screening by LC–MS/MS to confirm the presence of 3,4-HDMP and the absence of 3,4-DMP.

### 3,4-DMP Quantification

LC–MS/MS (Agilent) was performed as previously described (8) using an Agilent 1200 series LC and 6470 triple quadrupole tandem mass spectrometer. In brief, DMP was quantified using *m/z* 97.4→56.2 and qualified using *m/z* 97.4→70.2, and 3,4-HDMP used *m/z* 99.2→57.2. The same Phenomenex Kinetex biphenyl column (50 × 3 mm, 2.6 µm) was used, and a second Phenomenex Kinetex C18 column (50 × 3 mm, 2.6 µm) was also used to identify whether the stationary phase played a specific role in the chromatography. Perfluorooctanoic acid (0.01%) in water (phase A) and methanol (phase B) was used for quantification and the flow rate was optimized to 0.4 mL/min, with an injection volume of 1 µL. The gradient started with 20% phase B for 0.5 min and then increased to 80% by 4 min, held until 7.5 min and then returned to 20%.

### 3,4-DMP Recoveries from Soil

Oven-dried soil (2 g) was spiked with 3,4-DMP at five soil concentrations (0.1, 0.5, 1, 20, and 150 ng/g) in quadruplicate as previously described (8) in centrifuge tubes (15 mL) pre-treated with Coatasil™ and then moistened with water. Stocks of technical grade 3,4-DMP (20 µL) were spiked into the soil, mixed, and stored for one week at −20°C. After defrosting, tubes were spiked with 3,4-HDMP as an internal standard and then NaOH (0.1 mL, 5 M) and acetonitrile (5 mL) were added. The samples were mixed using a vortex mixer (10 min) and then centrifuged (5 min, 2100*g*). Enhanced Matrix Removal (EMR) polish (NaCl/MgSO4, 1:4, w/w) was added to remove water, and after mixing on a vortex mixer (1 min) and centrifugation (5 min, 2100*g*), the acetonitrile extracts were transferred to glass vials and evaporated under nitrogen (30°C). The residues were resuspended in methanol (300 µL) and centrifuged (5 min, 21 000*g*). Supernatants were recovered for LC–MS/MS analysis.

## Results and Discussion

### Optimization of Ion-Pair Conditions

**Figure 1.**
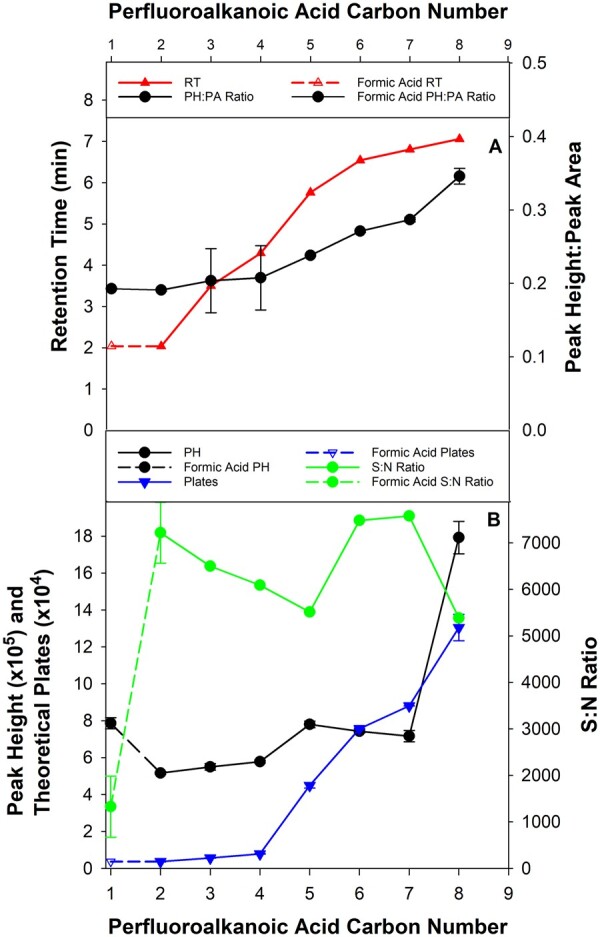
(A) Retention times and PH:PA ratios and (B) S/Ns, theoretical plates and peak heights for C2–C8 PFAAs compared to formic acid as mobile phase modifiers at a 0.1% concentration and a flow rate of 0.3 mL/min (mean ± SD, *n* = 3).

PFAAs from perfluoroethanoic acid up to and including perfluorooctanoic acid (PFOA) were tested as ion-pair reagents at a concentration of 0.1% (v/v). RT, peak height (PH), peak area (PA), PH:PA, theoretical plates (N) and the S/N were used to assess the effectiveness of ion-pair reagents to retain 3,4-DMP on the column, as well as their impact on peak shape and baseline noise. While perfluoroethanoic acid resulted in the same 3,4-DMP RT as formic acid, increasing the PFAA carbon number also increased the RT of 3,4-DMP from 2 to 8 min, presumably due to greater hydrophobic interactions between the ion-pair reagent and stationary phase (Figure 1A). The increased RT provided a greater opportunity to divert the LC flow containing dissolved minerals and SOM from the MS/MS spray chamber, while also potentially reducing suppression of ionization. Likewise, the PH:PA ratio increased with increasing carbon number compared to formic acid (Figure 1A), which occurred as a disproportional increase in PH compared to PA. The absolute PH of PFOA was more than double any of the other ion-pair reagents, but came at the expense of increased baseline noise, which decreased the S/N by approximately 25% (Figure 1B). Despite this reduction, PFOA resulted in an N increase of approximately 50% over perfluoroheptanoic acid, and a 35-fold increase over formic acid, making it the preferred choice for the ion pair (see supplemental information). Mobile phase ion-pair concentrations were measured using percentages in this study for preparation convenience. The molar concentrations were 27 mM and 16 mM for formic acid and perfluoroethanoic acid, respectively. As the molecular weights of PFAAs increase by 50 amu for each additional fluorinated carbon, the equivalent concentrations for the C2–C8 series tested decrease from 16 to 4 mM in an exponential fashion, which results in an insignificant bias. While the formic acid PH was 50% larger than the perfluoroethanoic acid PH (Figure 1B), formic acid was not capable of retarding 3,4-DMP on the LC column to any significant extent, and lowering the formic acid concentration to match the PFAAs would not change this inescapable fact.

**Figure 2.**
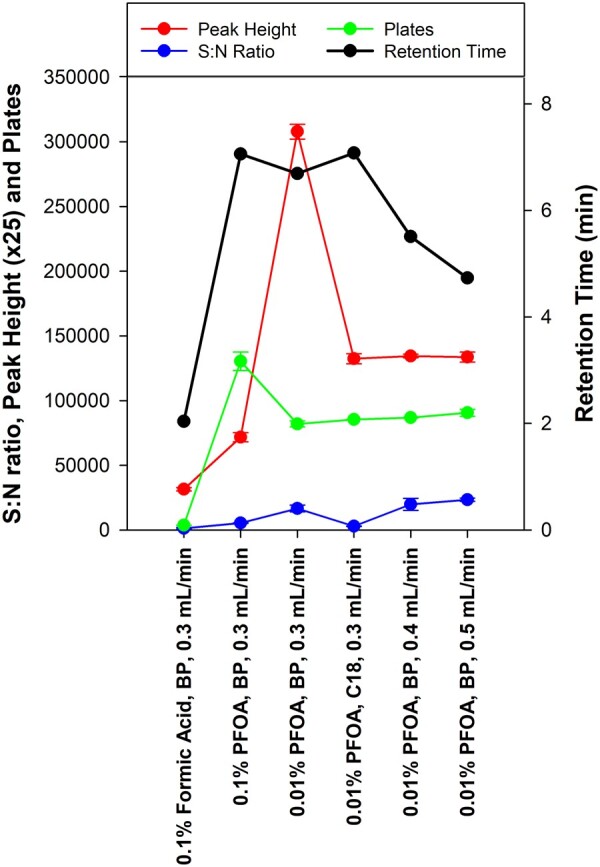
Comparison of formic acid with PFOA at 0.1 and 0.01%, and the effect of flow rate on column performance for 3,4-DMP elution (mean ± SD, *n* = 3).

Ion-pair reagents at concentrations of 10–100 mM are generally required for full column saturation, and concentrations of 0.1–10 mM have been reported as typical concentrations for analytical purposes (10), which would equal 0.004 to 0.4% for PFOA. However, lower concentrations of mobile phase additives are preferred where possible to reduce baseline noise, so the concentration of PFOA was reduced from 0.1% (4 mM) to 0.01% (0.4 mM) to identify impacts on baseline noise and ionization suppression. The RT reduced slightly from 7.06 to 6.69 min, and N decreased from 130 000 to 82 000 (Figure 2). However, the PH increase by more than 70% and the S/N increased by 200%, indicating improved ionization in the MS/MS and reduced baseline noise. To compensate for the decrease in N, the flow rate was increased from 0.3 mL/min, which decreased the RT but provided a slight increase in the S/N ratio, N, and PH. A flow rate of 0.4 mL/min was considered an appropriate compromise for conditions and provided a 5.51 min RT, which would still allow for extracted soil mineral salts and some SOM to be diverted from the MS/MS source. PFOA was only included in phase A (water) of the mobile phase and not in phase B (methanol) because its inclusion in phase B caused 3,4-DMP to elute almost a minute earlier, and it also caused 3,4-DMP to suffer a 30% reduction in PH. This resulted in extensive peak tailing, which was characterized by more than double the baseline peak width (see supplemental information). Baseline noise also increased considerably, which is a common observation for PFAAs (11–13). The resulting S/N was several orders of magnitude lower, further confirming pure methanol as a better option for the organic phase.

A new C18 column with the same dimensions, particle size, and core shell technology, and from same manufacturer as the biphenyl column, was used to determine whether minor stationary phase differences or the age of the column impacted ion pairing. Both phases rely on hydrophobic interactions, while biphenyl also offers pi bond interactions, which are unlikely to contribute to PFOA interactions. The RTs under both systems were the same, indicating that ion-pair reagents have the ability to not only improve retention, but provide a second life to an old column that had been subjected to more than 10 000 plant and soil extracts over several years. The results do not provide any greater clarity on whether ion pairs formed and these pairs moved along the column, displacing unused ion-pair reagent adsorbed to the stationary phase, or whether ion-pair reagent was bound to the stationary phase, thereby converting the column to an ion-exchange column.

### Recovery of 3,4-DMP from Soil

Soil was spiked with 3,4-DMP at five different soil concentrations and recovered using the synthesized 3,4-HDMP as an internal standard. Recoveries ranged from 102 to 107% (mean of 104.5 ± 1.9%) when using 3,4-HDMP as an internal standard (Table 1), indicating a slightly greater loss of the internal standard than analyte during extraction. Within and between batch variabilities were 3.1% and 4.4%, respectively, and were similar to the previously published values of 3.4 and 5.7%, respectively. Recoveries of 3,4-DMP in a previous study using the structural isomer 3,5-HDMP reported recoveries of 83.7 to 86.3% over a soil concentration range of 0.5–167 ng/g, indicating greater loss of analyte than internal standard (8). The results in the current study indicate greater similarity in extraction between 3,4-DMP and 3,4-HDMP, regardless of the soil concentration tested, and indicate that 3,4-HDMP is a more appropriate internal standard than those tested in previous (8) and current studies. When considering 3,4-DMP recoveries without normalization with the 3,4-HDMP internal standard, absolute recoveries ranged from 69 to 78%, with poorer recovery occurring at higher applied 3,4-DMP concentrations. This may be a result of a greater amount of 3,4-DMP sorption to less accessible sites on the soil at higher concentrations, but is unlikely to be saturation of the extraction solution given the relatively low soil application rates. Increased error in the absence of an internal standard reinforces its importance in multi-stage extraction methods where the likelihood of analyte loss is greater.

**Table 1.** Recovery of 3,4-DMP using 3,4-HDMP internal standard, and absolute 3,4-DMP recovery without the use of an internal standard

Soil 3,4-DMP concentration, ng/g	3,4-DMP recovery with 3,4-HDMP IS, %	Absolute 3,4-DMP recovery, %
0.1	107.0 ± 7.3	77.6 ± 7.5
0.5	103.7 ± 6.4	76.1 ± 5.0
1	105.2 ± 4.7	76.5 ± 4.8
20	104.9 ± 3.3	70.6 ± 7.0
150	101.8 ± 3.6	68.5 ± 6.3

The previously published method (8) used a 1 µL injection volume to report an LOQ of 0.5 ng/g, while the current method achieved 0.05 ng/g. Increasing the injection volume to 2 or 5 µL can improve this value slightly without any apparent peak broadening issues. The LOQ improvement in the current method is largely the result of improved chromatography resulting from the use of ion pairing to improve 3,4-DMP retention, and provides the analyst another order of magnitude.

## Conclusions

Extraction of pesticides from soil requires cleanup to remove organic and inorganic chemicals co-extracted from the soil. Rapid sample processing with limited or no cleanup requires improved separation of analytes from the matrix to prevent the matrix entering the MS/MS source, which can cause blockages, ionization suppression of analytes, and contamination of the ion source. Small polar molecules like 3,4-DMP can be difficult to retain on an LC column, making separation from a dirty sample matrix impossible. Addition of ion-pair reagents, such as PFAAs, to the mobile phase can improve column retention and, using adequate concentrations, can also give older LC columns a new life. The column used in the current study had more than 10 000 injections of plant and soil extracts and would otherwise have been discarded, but performed well when using PFOA as an ion-pair reagent. While using fluorinated chemicals is not ideal from the perspectives of waste and human exposure and potential corrosion of metal surfaces in the MS/MS, 3,4-DMP retention was increased, and peak shape and height improved dramatically. Ionization suppression by PFOA was minimized by only including it in the aqueous phase and allowing elution to occur with pure methanol. Narrowing the internal diameter and increasing the column length may provide further enhancements to what is already a sensitive technique.

## Conflict of Interest

All authors declare no conflict of interest.

## Supplemental Information

Supplemental information is available on the *J. AOAC Int.* website.

## Supplementary Material

qsac126_Supplementary_Data
