# Supplementary material for: Quantification of 3,4-Dimethyl-1H-Pyrazole Using Ion-Pair LC–MS/MS on a Reversed-Phase Column
Source: J AOAC Int. 2022 Oct 26;106(2):316–9. doi: 10.1093/jaoacint/qsac126 (PMC9978597; doi:10.1093/jaoacint/qsac126)
Supplement: qsac126_Supplementary_Data [file qsac126_supplementary_data.docx]

Supplementary Material


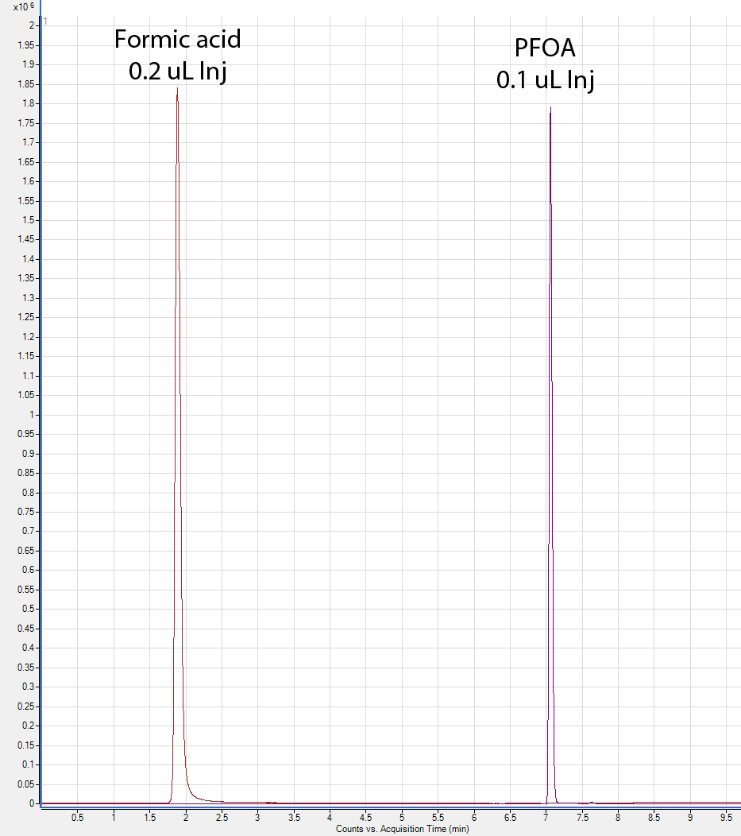


DMP using formic acid mobile phase, and DMP using perfluorooctanoic acid (PFOA) mobile phase, both at 0.1% and 0.4 mL/min. Half the injection volume resulted in a peak of similar height.


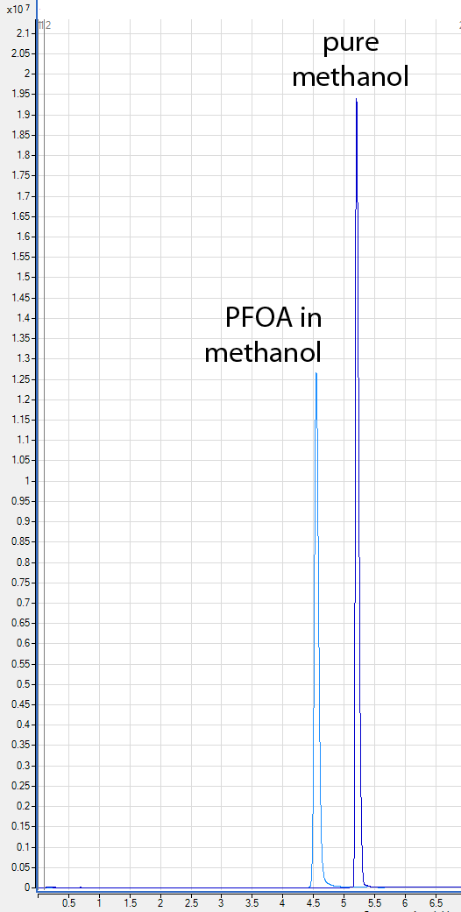


PFOA in the organic phase of the LC at 0.01% and 0.4 mL/min.
